# Work, identity and health

**DOI:** 10.1186/1745-0179-2-12

**Published:** 2006-05-31

**Authors:** Tom Fryers

**Affiliations:** 1 Visiting Professor of Public Mental Health, Department of Psychiatry, University of Leicester, UK

## Personal introduction

Having free-lanced as a public health physician for over a decade, with the inevitable variation in both the avaiIability and type of work, 'retirement' is, perhaps, a vaguer concept than for those with conventional full time jobs. In my middle sixties, I was drifting towards retirement, thinking that I should soon refuse any new work, when an incident occurred quite unconnected with work. Many have experienced being treated by someone with utter contempt, but a dramatic confrontation in which I was made to feel a worthless 'nothing' forced me to reflect on my immediate future, my social status in retirement, and the importance of work. I abandoned thoughts of full retirement and took up new research commitments, having concluded that, for me, it was important to be a 'something', whatever that 'something' was.

Since then, talking to many others about retirement, I have found both resonances and differences in their concerns and strategies approaching retirement, but many other issues concerning the status of work in our society have arisen. This paper was an attempt to explore these issues for the 2004 meeting of the European Association for Medicine of the Person, Drubeck, Germany. The Association derives historically from the work of Paul Tournier a Swiss general practitioner, whose approach as a Christian physician encompassed physical, psychological, spiritual and social elements [1,2].

## A. Work

### 1. The work ethic

Many 'Western' societies are very work-oriented cultures. Work is usually necessary to earn a living, and there are strong moral pressures to work deriving from our Christian heritage. Luther saw daily work as a vocation which we are bound to undertake under God, sharing in His work on earth. Calvinists believed that hard and successful work was a sign of salvation [3]. John Wesley, founder of Methodism, said, in 1746 [4] that we should "make our daily employment a sacrifice to God; to buy and sell, to eat and drink, to His glory." These attitudes became very powerful, especially in protestant communities, leading to the 'protestant work ethic', which both Max Weber and RH Tawney considered to underpin the rise of modern capitalism. Marx analysed the way in which work reflects social structures and argued that the way it is organised shapes both society and individual lives, but, whatever the context, the importance of work was emphasised [3].

Paul Tournier in 'Learning to Grow Old' [5] wrote; "In the West, almost everyone agrees in proclaiming in words, in theory, the supreme value of the human person. We quote ... 'Man is the measure of all things'. It is man, we say, who gives work its dignity, and not work which gives man his value. But in reality things are quite different. The general social atmosphere in which we are immersed from childhood, and which influences us without even our thinking clearly about it, teaches us the superior importance of work. Work, as a duty, is seen to be full of dignity, even if it is tedious or inhuman." We might note, in the UK parliamentary election of May 2005, the insistence of all three major parties that they serve the interests of 'hard-working families'!

Thomas Carlyle and John Ruskin, sharing that protestant inheritance, nevertheless gave a different emphasis, ideological and romantic. Ruskin believed that men should and could be happy in work "God intends no man to live in this world without working, but it seems to me no less self-evident that He intends every man to be happy in his work" [6]; Carlyle said "Labour is life" [7]. William Morris commended Ruskin for demonstrating "that it is possible for men to rejoice in their work" [8].

### 2. Work in society

The eminent sociologist Peter Worsley [9] wrote: "Work is central to our culture. When someone asks "What do you do?" they really mean "What work do you do?". When a woman is asked "Do you work?", what is meant is "Are you doing a paid job?" Yet many people without a paid job work at other kinds of productive activities." So we note that the comments above referred largely to paid work, and applied almost entirely to men; issues concerning women's work have changed greatly and are both varied and complex; they will be addressed later.

There are cultures which do not value work as highly as in Europe and America. Lee [10] quotes Kalahari Bushmen saying "Why should we work, when God has provided so many mongongo nuts?" But it has been generally held in 'western' societies that people should work hard and conscientiously at whatever job they have, both to earn money for their families, and to serve the community. However, it was also a common assumption that material improvement, accumulation of wealth, increased social status and influence, that is, worldly success, was a sign of God's blessing on you and your work. One still hears echos of this, alongside individualistic ideologies and wide disparities in wealth.

Preparation for a society in which work-values predominate has become the principle ground of education. The voices of those who argue for classical education, or education for life, or education for increased leisure, or even education for the 'global village', are lost in the clamour of those who say that education should be for 'the world of work'. Because it does not do this very well in a constantly changing 'world of work', governments frequently change the system, and teachers are unclear what is expected of them. Children are expected to find their adult identity in work, but are not well prepared to do so. And their more fundamental needs – 'education for life' perhaps, will not have been met. Perhaps this is part of the explanation of anti-social behaviour in the young; having no clear identity, no clear status and no clear role in the adult world?

Work is perceived, therefore, as not only providing an income, but giving social legitimacy to our lives. For many, it may be the principle source of personal identity, mediating the sense of being a valued person necessary for self-esteem. A profession or trade gives us an identity – as a doctor, a teacher, an engineer, a motor mechanic, a secretary, an electrician, and so on. A particular job, independent of profession or trade, may give us an identity – as a consultant, a manager, a director, a foreman, a work-team leader. Or we may derive a sense of identity simply from being a worker, a participant and contributor; people who cannot work, or cannot find work in our society may understandably envy even this identity.

## B. Attitudes to work

### 1. The value of work

Our societies have changed substantially in recent decades, and continue to change, not least in attitudes to work, but attitudes are also very varied. Some see work as an unpleasant chore, to be undertaken as little as possible only because you need money, and to be given up as soon as possible. Some will complain about aspects of their particular job, but see work in general as a good thing, without which life would lose a lot of its meaning. Others may see work – at least, their work – as the main source of life-satisfaction, deeply rewarding, important for the community, and hard to relinquish.

Most of us will have shared these attitudes at different periods in our careers, or even concurrently about different aspects of our work. Whatever the justified complaints of doctors, nurses, teachers and other professions, we are privileged to have such professions, providing jobs with intrinsic intellectual, emotional, personal, even spiritual satisfactions, with social respect, and, usually, more than adequate pay.

For some jobs there is little satisfying in the tasks to be done, but work has other things to offer, without which people can feel lost, useless, un-valued, of no clear identity.

Whatever the job, it can give a sense of belonging, of being a contributor; an important part, however menial, of an organisation with a bigger purpose, a valued part of society. A nice example is the care-taker/cleaner portrayed by Gervase Phinn in his fictionalised accounts of a Yorkshire education service [11].

Work can provide a structure for the day, week and year without which life just drifts by, a commitment to yourself and others, a purpose for getting up in the morning and going to do something. Our local sheep farmers, in spite of the current dis-satisfactions with farming, are time-tabled by their flocks.

Work can provide the satisfaction of doing something well, whatever it is, of pride in your work, of being a 'craftsman', of seeing a good end-product. We have recently experienced this with a local builder, joiner plumber and electrician, all of whom had a deep commitment to craftsmanship.

Work can provide friends, a social group to belong to, companionship, shared lives in joys and sorrows, support when suffering or in need. My father, with a job well below his potential, found this in the 'Sports and Social Club'.

Any or all of these can play a major role in fostering self-esteem, an essential ingredient of good mental health, and closely tied up with our sense of being a valued person, of having a personal identity. Not everyone finds this in work; there are other sources of a sense of self-worth, but for many individuals, and for society in general, work is one very important source of self-esteem and personal identity. We need, therefore, to consider the situation of those who do not or cannot work, but first, we will look briefly at those who work to excess.

### 2. Workers to excess

There are several types of excessive worker, in all of whom there may be substantial stresses related both to working and to not working. There are those who work for themselves, running their own business, who work very long hours to make the business a success, to earn more, or because they see the business as dependent on them and they cannot let go. They may be identified almost wholly by their work and may have little other life. Sickness or retirement may be a huge loss, for they have no other investment for their lives. PG Wodehouse portraits several examples of retired business men, very successful, very wealthy, desperately taking up a hobby with the same obsessive approach that characterised their business life [eg [12]].

There are those who are employed but are so involved in and committed to their job that they cannot keep away from their work. Some academics and some health workers are like this. In extreme cases this may create stresses in both the worker and their family, and may make sickness or retirement appear catastrophic. However, it may be possible to perpetuate their identity, for example by writing.

These groups elect to work excessively; a third group does not really choose to do so. They work very long hours, far more than they are officially paid for, because it is expected of them by the culture of the firm or wider society, and because they share a perception that keeping their job, gaining promotion, or moving to a better job, demands 150% commitment to work, and nothing else ranks of similar importance in their lives.

This attitude seems to be increasing in the UK, and perhaps elsewhere in Europe. I am told by my youngest son in Washington DC that it is very common, even characteristic of many jobs in the USA [13]. In this situation, people may be forced by societal pressures to identify as a 'worker', not necessarily in any particular trade or profession, equating long hours with importance and success. Perhaps for such workers, if financially secure, retirement (and even sickness), and relinquishing their worker identity may be a great relief.

A fourth group, with commitments beyond their means feel forced to work extra hours or additional jobs in order to cope financially. They may be on low wages, supporting uneconomic family members, or a family life-style incurring substantial debts. Both can be very stressful situations, and work may seem little more than slavery.

## C. People not in paid work

### 1. Those who will not work

Society exhibits ambivalent attitudes towards the few who are wealthy enough not to work – they are both envied and resented, even though some fill their lives with 'good works' and are highly respected. But those without wealth who are perceived as unwilling to work are denigrated as 'loungers', 'loafers', 'work-shy'. There are, no doubt, adults who are fit and well and lazy, who will not work if they can get away with it, though I believe they are few. However, it is not always easy to distinguish these from those who have genuine reasons for not working – physical ill-health, mental ill-health or serious social stresses – or who cannot find work.

### 2. Those who are unemployed

Those who cannot find work, the unemployed seeking work, are put under increasing pressure. Many, for example in the North-East of England, have had to leave their families to find work in other regions; they cannot afford to move house, and, in any case, jobs have little security. These men (mostly) are working, but the stresses on them and their families are huge. It is not surprising if marriages break down and families split up. But for many men, unemployment is the worst option, bringing low income, low status, loss of purpose, loss of dignity, loss of identity.

The relationship of unemployment with illness is well established, though physical illness is likely to be more commonly cause than effect. It is likely to be a mixed picture with respect to depression and anxiety, the 'common mental disorders'. Following a recent review of all large-scale population studies since 1980, we can be confident that there is a substantial excess of these disorders associated with poor educational background and low material resources, as well as unemployment [14,15]. Many Family Doctors will be well aware of these things in their daily practice.

### 3. Sickness absence from work

The confusion of refusal to work, inability to find work and inability to work is very damaging, because there are many who can't easily work on account of acute illness, long-standing sickness, or serious disability, not all of which is obvious to the casual observer. At this point doctors play a major part in helping to distinguish reasons for not working, as, in many societies, they have a legitimising role for sickness absence from work or receipt of sick pay and disability pensions. The numbers involved are large. For example, in Sweden, in 2000, of 5.6 million adults of working age, 62.4% were in work, and only 4.7% were unemployed; but 11.8% were on sick-leave, and 21.1% were in receipt of disability pensions [16]. In the UK, in the three months March to May 2004, 1.7 scheduled working days were lost to sickness absence, involving 2.9% of all UK employees [17].

Sickness certification is the means by which society formally permits people to be not working without loss of dignity or loss of identity, but they have to fulfil certain conditions as part of the 'contract' with the community [18]. They must allow their claim of illness to be verified by a doctor, must refrain from work, and must behave appropriately. As all doctors know, certification is important but inherantly problematic. Short periods are usually excused verification, and specific industrial diseases (accidental injury, certain cancers, pneumoconioses, etc) raise different issues which will not be dealt with here.

But, sick-leave from work is often a complex clinical, personal and social phenomenon, the presenting complaint not necessarily being the sole or principle problem. We know for example, that sickness absence increases with adverse conditions at work [19]. These include pressure to increase the pace of work, conflicting demands, and low control over your own work. (It is interesting to note Ruskin's three conditions for being happy at work: they must be fit for it; they must not do too much of it; they must have a sense of success in it [6]). Problems outside work, in families and other relationships, may be important. However, if approached holistically, in the manner of Paul Tournier, these situations have positive potential for medical practice, requiring careful physical, psychological and social diagnosis, and prescription of a wide range of appropriate therapies, and personal and social interventions.

### 4. People with disabilities

Very few people with disabilities cannot work in any way; most, of course, have significant abilities, and want to work as far as they are able. But their ability to work often depends as much upon the environment, practical circumstances and attitudes of others, as upon their own limitations. We have gone some way to adapting work situations to permit people with limited sight, hearing or mobility to work, but there is much more to do.

Identity is a key issue, with two facets. First, they may suffer discrimination as non-workers, lacking the possibilities of identity from a profession, trade, job or worker-status. Second, they are labelled with an identity as 'disabled', or even as the type of disease or disability they suffer from. The worst stigmatising terms are often used in society as terms of general abuse, but it is also common to refer to people as 'a diabetic', 'an epileptic', 'a schizophrenic', and so on. It must be very hard to live with your identity as a disease category, though, of course, there are many individuals who have overcome all these things with heroic fortitude and great success.

### 5. Women

I pointed out earlier that most of the literature relating to work applies to men. From a current view-point, it is amazing how strong was the historical assumption that, in relation to paid work and economic activity, women could largely be ignored. Even Tournier's book of 1971 [4] seems to assume 'traditional' women's' roles. But worker-identity remains problematic for many women. Except where paid to undertake it for someone else's family, the traditional house-wife and mother role, whilst arguably the most important in all society, has never been awarded the status of a job, even less a career or profession, in spite of recent attempts to do so. Research classifications still cannot cope with it; traditionally married women were classified by their husband's job.

In my parents' generation, it was probably still 'normal', though by no means universal, for most women to expect no profession or career except that of housewife and mother. My mother was withdrawn from school by her father to look after the family on the grounds that a girl did not need education; she never 'worked', and always felt something important had been lost. This attitude towards women's work can still be found among men. Among women it is often implied: how often have you heard "I'm only a housewife"; no job, no status, low self-esteem. Yet, raising a family and running a household may be very demanding and, especially with larger families, an excellent training in organisational and managerial skills. We might do better to recruit some of these experienced 'housewives' into business and public service management, than many of the men we employ.

Of course, many of my generation have worked when their children were older, but, in spite of equal opportunities legislation, women get less top jobs, often get less pay than men doing the same job, and predominate in part-time jobs, which often carry poverty wages. Can this partially explain why women have more of the common mental disorders than men? In the British National Psychiatric Survey of 2000 [20], women were twice as likely to have obsessions, somatic symptoms, compulsions and phobias, and almost one and a half times more likely to suffer symptoms of fatigue, to have problems with sleep, and to have a total of neurotic symptoms above case-threshold. Mixed anxiety and depressive disorders in the age group 16–64 were 19% for women, 13% for men.

Of course, nowadays, many women are training and pursuing careers of all sorts. As we well know, many are postponing (where not eschewing altogether) having children until well into their thirties, with long-term consequences which may not be entirely satisfactory. Their 'worker-identity' may be a real gain, with improved self-esteem, but surely we still need to find a way of recognising the 'housewife and mother' role, the 'domestic manager' role, and investing it with a clear, high-value identity and due status, especially as many women find themselves doing 'double duty' – working both outside and inside the home!

### 6. People who have retired

The idea of retirement is relatively modern and is characteristic of industrial societies [21]. Pre-industrial societies always had a category of people called 'old', for whom the role expectations were different from those of younger adults. But new roles, usually requiring less physical strength, were aquired gradually and at no specific age, a transition to different but equally valued roles – men might no longer be hunters and farmers, but elders and priests. Although retired people in modern industrialised societies have many roles, and undertake many responsibilities, such as looking after grand-children, these may be considered rather trivial, and not highly valued except, perhaps, by the parents of those children.

The situation is likely to change. Where unemployment is low, as in the UK, we are short of workers, short of skills and experience in many trades and professions, and need people to stay in work longer. People over 65 are now much fitter and healthier, and are mostly able to work; many want to work, though not necessarily full-time. Yet ageist attitudes persist; there is widespread prejudice against employing older workers, even in their fifties. With the increase in older people, the financial burden of pensions has become a big political issue, and many private sector pension schemes have failed, with workers losing their pensions.

Although the traditions and social arrangements for retirement vary between countries, the main issues are generally the same. Many perceptions of retirement for men are very negative; "a first step towards social dependence" [22], of which the pension is a symbol. Media reports referring merely to 'a pensioner', with no name, no personal identity, tend to reinforce negative images. Like 'the elderly', it is not really pejorative, but it does imply dependence. It is a non-identity, symbolic of low personal value.

In Townsend's classic 1954–55 study of working class Bethnal Green [23], although poor pensions were a problem, men complained just as much of boredom and a sense of uselessness – of being unwanted. He reported "Among the retired, there was scarcely a single person in favour of retirement." I think that this would not be as true now in any group; many have good pensions, and opportunities for alternative activity have increased dramatically for most people. But for many others, it remains an experience of loss, including a profound loss of identity.

Tournier [4] recognised this common sense of loss with its consequences for ill-health and unhappiness in older age. He advocated deliberate preparation for retirement, now more common, and the attention at earlier ages to interests outside work which will sustain life in retirement. He thought that unstructured hobbies did not go far enough, and proposed the idea of a 'second career', by which interests and activities in retirement should be structured, should have "coherence and continuity", like a previous career, so that it resembles 'work'. Nowadays, it might be a third, fourth or fifth career, but the principle is the same.

Some people do obtain paid work after retirement, though often part-time, and often of a lower status than their previous work. Some can use their skills and experience in consultancy, teaching or writing related to their main career, and retain their work-related identity. Some, especially retiring early, re-train and take up a new paid career in a more satisfying field than their previous one. But many, assuming an adequate pension, do not look for paid work, but, very much as Tournier suggested, pursue a new career in voluntary work with NGOs. In the UK at least, NGOs are largely dependent upon an army of retired people. To be chairman or a trustee of a major charitable organisation requires abilities, skills and experience akin to those needed for senior positions in private business or public service, and carries similar roles, responsibilities, commitments and risks.

These positions carry some status in society, but I do not think that they confer a recognisable work-type identity. Perhaps those that undertake them for a long period are people who do not crave work-related identity, but find their personal identity from other sources. They include many women, who may have been denied work and career identity in their main adult years, but have achieved a satisfying sense of personhood at a deeper level in terms of relationships, family, and community service.

Since each country has an official 'retirement age' when pensions will normally begin, and when not-working is accepted without stigma, 'early retirement' is likely to have some special features. It became common in the UK about twenty-five years ago, partly in response to high levels of unemployment, and the rate of technological change. It was sometimes a matter of individual choice, encouraged by generous early pension arrangements. But it was often forced on people through redundancy, and 'ageism' among employers, which precluded people in their fifties getting new jobs. Some retired early in response to poor health; in some cases a partial reason, but conveying legitimation. For society there were huge losses in skills and experience, now openly regretted in many fields. Also regretted is the huge extra financial burden of pensions on companies and public institutions, for no productivity. Retirement at 50 incurs an expected pensioned life of 30 years or more.

Physical health problems may mostly precede early retirement, but mental health problems may be either causes or consequences of retirement. There is evidence of causes – 20% of early retirements from the UK National Health Service are for psychiatric reasons [24]. There is also evidence of consequences – involuntary early retirement appears to increase the prevalence of the common mental disorders, but planned, voluntary retirement appears to reduce them [25,26].

Data from the British National Psychiatric Survey of 2000 are very interesting [27,28]. They showed a significant drop in prevalence of the common mental disorders (mostly depression, anxiety or both) at the age of 65 for men, but not for women. For men, total symptom counts of 18 on the Clinical Interview Schedule (CIS) were 7.3% at age 60–64 and 1.5% at age 65–69. 'Depression', was 3% at 60–64, and 0.3% at 65–69. It is not a cohort effect (from analysis of the 1993 and 2000 surveys together), and no variables explain it statistically other than age. Men who retired early had high rates, indeed higher rates than employed men, until 65, when they suddenly achieved similar low rates. Men still employed after retirement age not only had low rates then, but had relatively low rates in their fifties and early sixties. But the lowest rates were for those who retired at 65. For women, the rates were higher than men at all ages, peaked at age 50–54, then slowly diminished with no large reduction at either age 60 or 65.

These are British figures; it would be interesting to have this replicated in other countries, but few have appropriate surveys. It seems that early retirement is bad for men, but retirement at the age legitimated by society is very good for men. Why? Perhaps legitimation is the secret; at age 65 perhaps, the generality of men can relax, no longer needing to justify themselves as workers; can, perhaps, 'be' more than 'do'. And, perhaps, people like me, who make such a fuss about retirement, are out of step, and need to learn something about personal identity which has nothing to do with being 'something' in work.

## D. Conclusion: identity – what am I? – who am I?

Who we are and what we are, are very important questions for all of us, with social, psychological and spiritual dimensions. Without a clear sense of personal identity, it is difficult to have the self-esteem we need to function well as independent people in inter-dependent society. Without a clear sense of personal identity we are vulnerable to psychological injury, at risk of anxiety and depression, and social disengagement. Without a clear sense of personal identity we cannot easily respond to love with love, to accept forgiveness, to start again after failure.

For many people, work, a job, a profession or a trade, provide an important source of personal identity; for some it may be the only significant source. Even for those for whom this is minimal, being a worker is important for the other benefits work mediates. Both before and after retirement age, continuing some work, paid or not, perhaps part-time, but definitely 'work', may be important both for individuals and the community. And for those with specialist skills and experience, is there not also a moral duty to continue to make a contribution for as long as it is needed and remains appropriate, whether financially rewarded or not?

However, work as the source of our identity is fundamentally inadequate, because few of us can claim that identity for ever. Sickness, disability, redundancy, retirement, age, all threaten an identity built upon work. But it is not the only source; we all do have other identities. We are sons and daughters, mothers and fathers, grandmothers and grandfathers and great-aunts, friends and neighbours; identities, built upon personal relationships, and surely more fundamentally important.

Ultimately personal identity and self-esteem are closely bound up together, and derive from a sense of personal value, of personal worth, of being needed, of being loved for what you are, not just for what you do. This is true health and wholeness, and, no doubt, much depends upon our experience of parenting as children. It is also a spiritual issue. Christians, and others of faith, will claim the ultimate value of a human being loved by God and therefore of infinite worth; others will be satisfied with a non-religious belief that each individual human being has intrinsic equal and great value.

Personally, perhaps I should think again about retirement, and accept my rapidly developing identity as a grandfather; and maybe mongongo nuts will grow in the greenhouse.

## Note

The first version of this paper was given to the 56th 'réunion internationale de Médecine de la Personne' in Drübeck, Saxony, Germany in August 2004.
